# Genome-wide association mapping of resistance to a Brazilian isolate of *Sclerotinia sclerotiorum* in soybean genotypes mostly from Brazil

**DOI:** 10.1186/s12864-017-4160-1

**Published:** 2017-11-07

**Authors:** Wei Wei, Ana Carolina Oliveira Mesquita, Adriana de A. Figueiró, Xing Wu, Shilpa Manjunatha, Daniel P. Wickland, Matthew E. Hudson, Fernando C. Juliatti, Steven J. Clough

**Affiliations:** 1 Department of Crop Sciences, University of Illinois, Urbana, IL 61801 USA; 2 Universidade Federal de Uberlândia, Umuarama campus, Uberlândia, MG Brazil; 3 United States Department of Agriculture, Agricultural Research Service, Urbana, IL 61801 USA

**Keywords:** GWAS, Genotype-by-sequencing, SNP, Marker, White mold, QTL, GAPIT, CMLM, FarmCPU

## Abstract

**Background:**

Sclerotinia Stem Rot (SSR), caused by the fungal pathogen *Sclerotinia sclerotiorum*, is ubiquitous in cooler climates where soybean crops are grown. Breeding for resistance to SSR remains challenging in crops like soybean, where no single gene provides strong resistance, but instead, multiple genes work together to provide partial resistance. In this study, a genome-wide association study (GWAS) was performed to dissect the complex genetic architecture of soybean quantitative resistance to SSR and to provide effective molecular markers that could be used in breeding programs. A collection of 420 soybean genotypes were selected based on either reports of resistance, or from one of three different breeding programs in Brazil, two commercial, one public. Plant genotype sensitivity to SSR was evaluated by the cut stem inoculation method, and lesion lengths were measured at 4 days post inoculation.

**Results:**

Genotyping-by-sequencing was conducted to genotype the 420 soybean lines. The TASSEL 5 GBSv2 pipeline was used to call SNPs under optimized parameters, and with the extra step of trimming adapter sequences. After filtering missing data, heterozygosity, and minor allele frequency, a total of 11,811 SNPs and 275 soybean genotypes were obtained for association analyses. Using a threshold of FDR-adjusted *p*-values <0.1, the Compressed Mixed Linear Model (CMLM) with Genome Association and Prediction Integrated Tool (GAPIT), and the Fixed and Random Model Circulating Probability Unification (FarmCPU) methods, both approaches identified SNPs with significant association to disease response on chromosomes 1, 11, and 18. The CMLM also found significance on chromosome 19, whereas FarmCPU also identified significance on chromosomes 4, 9, and 16.

**Conclusions:**

These similar and yet different results show that the computational methods used can impact SNP associations in soybean, a plant with a high degree of linkage disequilibrium, and in SSR resistance, a trait that has a complex genetic basis. A total of 125 genes were located within linkage disequilibrium of the three loci shared between the two models. Their annotations and gene expressions in previous studies of soybean infected with *S. sclerotiorum* were examined to narrow down the candidates.

**Electronic supplementary material:**

The online version of this article (10.1186/s12864-017-4160-1) contains supplementary material, which is available to authorized users.

## Background

Many advances are being made in soybean (*Glycine max* (L.) Merrill) breeding that have been reflected by continual increased production worldwide [1]. For example, in the US there has been a steady approximate 1.9% average increase in yield (kg/ha) per year from 1960 to 2015 (http://usda.mannlib.cornell.edu/MannUsda/viewDocumentInfo.do?documentID=1290/). However, although yields are increasing, yields are still vulnerable to a variety of environmental restrictions, such as the constraint caused by the constant attack by pests and pathogens. The pathogen *Sclerotinia sclerotiorum* (Lib.) de Bary is one such yield-limiting pathogen, causing the disease Sclerotinia Stem Rot (SSR), and causing considerable damage in regions that have had several weeks of cool wet weather during the flowering period [2].

Resistance to *S. sclerotiorum* is not complete in most dicotyledon crops such as soybean. Instead, resistance in soybean to SSR is only partial, with multiple alleles providing a small amount of enhanced defense. Due to this lack of adequate resistance in commercial varieties, and the heavy influence of weather conditions, losses due to SSR can be zero one year, but then account for nearly half of all disease-associated losses the next, as seen in Iowa 2003 and 2004 [3]. Even though complete resistance to SSR is currently not available for soybean, there are clear differences in the susceptibility of tested genotypes [4–11]. And although not ideal, partial resistance to SSR does increase the yield potential, as slowing of the disease progression can be successful enough to allow plants to recover with minimal damage, especially if the weather changes to be more favorable to the plant, and less favorable to the pathogen.

Molecular studies looking into genes that are differentially expressed during SSR development in soybean identified thousands of genes responding within the first 24 h [12, 13]. These results, in addition to those of physiological and biochemical studies of *S. sclerotiorum* infection [14–16], show that *S. sclerotiorum* – plant molecular interactions are very complex, involving numerous pathogen-released proteins and oxalic acid for the plant to cope with [17]. The complexity of SSR partial resistance has led many researchers to use QTL analyses to identify markers associated with SSR resistance. Some early QTL mapping studies used biparental populations with limited genetic variation, or with populations of limited size [9, 18–21]. But due to limits of mapping studies involving only a few genotypes, and due to the subtle effect of these QTLs, it has been a challenge to find markers consistent enough to be satisfactorily applied to marker-assisted selection on a commercial scale.

In recent years the use of next generation sequencing technologies has led to a drastic reduction in the cost of genotyping, facilitating the identification of vast numbers of SNPs from large numbers of genotypes that can then be used for more accurate association mapping [22, 23]. The technique of genotype-by-sequencing (GBS) is one such approach to rapidly and cheaply produce a large number of single base polymorphisms (SNPs) based on the comparison of restriction fragment sequences that are held in common within the population [24, 25]. In this sense, studies of genomic association, proposed by Meuwissen et al. [26], are analyzed based on the evaluation of a large number of markers widely distributed throughout the genome. This type of association mapping involves the search for genotype-phenotype correlations in unrelated individuals and is often faster and more profitable than traditional biparental mapping [27]. Genotypic and phenotypic data are collected from a population in which kinship is not strongly controlled by the researcher, and correlations between genetic markers and phenotypes are sought within this population. In this context, the Genome Wide Association Studies (GWAS) may be a promising strategy for the identification of QTLs for genes of interest. Several GWAS reports have been published recently on soybean response to *S. sclerotiorum*, highlighting the common view that GWAS is a valuable approach to identify key genes and regions providing some enhanced resistance to this importance disease. Using GWAS to look at soybean response to SSR, Iquira et al. [28] found SNPs of high significance on chromosomes 1, 3, 8, and 20 with the strongest being on 1; Bastein et al. [29] found significance on chromosomes 1, 15, 19, and 20 with the strongest being on 15. By measuring chemical changes to pigments that might be associated with SSR resistance [30], Zhao et al. [31] found significance on chromosomes 6, 10, and 13 with the strongest being on 13. Of these two pathogen-based studies conducted by the same lab [28, 29], only two of the six QTL were identified in both studies, highlighting that conducting only one GWAS is not 100% effective in identifying all the variable nature of the SSR disease interaction, and that there is a need for additional analyses to identify more SNPs that are significantly associated with enhanced SSR resistance.

In this manuscript we present the results of another GWAS of soybean response to SSR, with a focus on genotypes from three different SSR resistance breeding programs in Brazil, and with the use of a Brazilian isolate for inoculations. Previously published SSR GWAS studies were conducted on genotypes largely used in breeding programs in Canada, US, and China, and with pathogen isolates collected in the Northern hemisphere [28, 29, 31]. Additionally, we compared significance using the GWAS analysis methods CMLM [32] and FarmCPU [33], as these programs handle testing markers, population structure, and kinship differently. The results provide soybean breeders with additional SNPs that they can have high confidence are associated with enhanced resistance to SSR and that could be used in marker-assisted selection.

## Methods

### Plant material, inoculation, and phenotypic scoring

Soybean [*Glycine max* (L.) Merrill] plants were grown in 500 ml plastic cups filled with Bioflora® (Bioflora LTDA-ME, Prata, MG, Brazil) organic plant growth substrate based on pine, other natural fibers, minerals and nutrient enriched, in a greenhouse at approximately 25–30 °C under natural light cycle in Uberlandia Brazil from December 2015 through February 2016 (approximately 12 h days). At least nine seeds were planted for each genotype. Soybean genotypes originated from the breeding programs of FT Sementes (Ponta Grossa, PR, Brazil), Tropical Melhuramente & Genetica (TMG, Cambé, PR, Brazil), or the Federal Universidade de Uberlandia (MG, Brazil), as well as various plant introductions (PIs) of the Embrapa Soja collection chosen from the literature. Immature shoot tips were removed at the V2 stage, and within 2–4 days, the plants were taken to the lab and cut just below the second trifoliate node. The freshly cut stems were inoculated with 2–3 day old *S. sclerotinorum* cultures of isolate Jataí [11] grown on potato dextrose agar plates for cut stem inoculation, a method shown to have low variability [34], with mycelial plugs picked up with a 200 μl pipette tip, similar to the straw test [35]. Plants were placed in an 18–20 °C, low light, growth chamber immediately after inoculation. After 4 days incubation, plants were phenotyped by measuring the length of necrotic lesion in centimeters.

### DNA extraction, GBS library construction and sequencing

Total DNA was extracted using a CTAB based method as previously described [36]. DNA integrity was verified on an agarose gel. Samples that were determined to be intact and of high quality, were pipetted into 96 well plates. Aliquots were removed to new plates to quantify with picogreen (Molecular Probes, Eugene Oregon, USA) on a Synergy HT (BioTek, Winooski, Vermont, USA) microplate reader. Based on both DNA amounts and plant samples, 352 samples were chosen, together with 32 controls (10 water blanks and 22 random repeats) for GBS library construction. GBS library construction was based on the method described in Poland et al. (2012) [25]. In summary, after adjusting DNA concentrations to be approximately 50 ng/μl, five microliters (250 ng) were pipetted into a new set of 96-well plates containing 2.5 μl 0.1 μM specific DNA barcoded HindIII adaptors. Pretesting of restriction enzyme pairs PstI-HinP1I, PstI-MspI, PstI-MseI, HindIII-HinP1I, HindIII-MspI, and HindIII-MseI indicated that the HindIII-MseI pair gave the best digestion results of having an even smear, with a broad peak near 300 nt (Additional file 1: Figure S1). Samples were therefore restriction digested using 1 U HindIII and 1 U MseI in a buffer mix at 37 °C two hours, followed by 80 °C for 20 min (all enzymes and buffers used for the GBS library construction were purchased from New England Biolabs, Ipswich, Massachusetts, USA). Following digestion, a common MseI adaptor was added, in addition to 40 U T4 Ligase and 1 mM ATP and 2× Cutsmart buffer were added, and the samples incubated at 25 °C for 2 h, followed by a 65 °C incubation for 20 m. After ligation, 8 μl of each well from each row were collected in a strip of 8 PCR tubes. Then 25 μl from each of the tubes of a strip were transferred to a 1.5 ml microfuge tube, totaling 200 μl that originated from one 96-well plate. Then 50 μl of samples were cleaned using Agencourt AMPure XP beads (Beckman Coulter Life Sciences, Indianapolis Indiana, USA), dried, and suspended in 15 μl a resuspension buffer. PCR enrichment was performed using master mix containing Illumina primers and NEB Phusion Master Mix. The following PCR settings were used for the enrichment reaction: 98 °C 30s, 15 cycles (98 °C 10s, 68 °C 30s, 72 °C 30s), 72 °C 5 m, 4 °C forever. After PCR, samples were purified using the Agencourt AMPure XP beads, and then pooled such that all the wells of a single 96-plate were now represented in a single microcentrifuge tube, and run on a Bioanalyzer 2100 (Agilent, Santa Clara, CA) using a DNA7500 chip to verify correct size, general success of amplification, and estimation of DNA amounts. Concentrations were adjusted to have approximately 10 nM DNA in 10 mM TRIS-HCl, 0.05% Tween-20 and run on a single lane of an Illumina HiSeq4000 using a HiSeq SBS sequencing kit version 4 at the Roy J. Carver Biotechnology Center on the University of Illinois (Urbana, IL) campus. The Fastq sequence files were demultiplexed with bcl2fastq v2.17.1.14 conversion software (Illumina).

### Processing the data from the Illumina sequence readings and selection of SNPs

The TASSEL 5 GBS v2 SNP-calling pipeline [37], together with the soybean reference genome assembly, Gmax_Wm82.v2 [38], were used for SNP identification. Prior to running TASSEL, the adapter sequences were removed from reads using the software Cutadapt [39]. To run the TASSEL pipeline, the default settings were used, except for the following parameters: minimum base quality score (mnQS 20), minimum mapping quality score (minMAPQ 20), and kmer length (kmerLength 80). The alignment step was performed using BWA-MEM [40] with default settings. SNPs with more than 50% missing data were removed, as were genotypes with more than 75% missing SNPs, prior to the imputation step, which was accomplished using Beagle 4.1 [41] with the parameter window = 1000, overlap = 200, and ne = 1000. After imputation, SNPs or genotypes with higher than 10% heterozygosity were removed from the dataset.

### Genome-wide association analysis

For the association analysis of SNPs to phenotypes, the compressed mixed linear model (CMLM) was selected and performed within the GAPIT package of R, which assigned similar individuals into 259 groups to estimate the kinship matrix [30, 39]. The reduced kinship matrix and three principal components (PCs) generated from principal component analysis (PCA) (Additional file 2: Figure S2) were included in the model to control population structure and individual relatedness. A batch effect was also included in the model as a covariate to control for possible variation between different phenotyping dates. The SNPs with a minor allele frequency (MAF) higher than 0.01 were used to estimate the population structure and the kinship. Only SNPs with a MAF higher than 0.1 were used for association tests. The cutoff of significant association was a False Discovery Rate (FDR) adjusted *p*-value less than 0.1 using the Benjamini and Hochberg procedure to control for multiple testing [40].

Another computational method named FarmCPU [31] was also implemented to do an association analysis, which separated the mixed linear model into a fixed effect model and a random effect model to reduce false negatives that might result from confounding population structure, kinship, and SNPs. The same batch effect and three PCs generated from GAPIT were included as covariates. Likewise, only SNPs with a minor allele frequency higher than 0.1 were used for association tests, and the cutoff for significant association was a FDR-adjusted *p*-value less than 0.1.

In addition to the two main models described above, three other models were also performed to test marker-trait associations. They were a naïve model without any control for population structure, a general linear model (GLM) with three PCs, and a mixed linear model (MLM) with three PCs and a kinship matrix without any compression.

### Linkage disequilibrium (LD) analysis

A function built into the TASSEL 5.0 program was used to determine squared allele-frequency correlations (r^2^) between pairs of SNPs with MAF greater than 0.1. The local LD was viewed, and LD plots were built, using Haploview 4.2 [42]. The LD blocks were defined with the Confidence Interval methods and the default parameters [43]. The LD decay plot was built based on the r^2^ values and distances between each pair of SNPs. To calculate the physical distance of LD decay (r^2^ < 0.2), a nonlinear model was used to estimate the expected values of E(r^2^) [44, 45].

## Results

### Phenotyping

Plants that germinated and grew healthily were used for DNA extraction and phenotyping. Most (108 of the 352 total utilized) genotypes were represented by nine plants, 86 samples had eight plants, 52 had seven, 48 had six, 26 had five, 20 had four, and only eight genotypes relied on just three plants. Following stem inoculation with *S. sclerotiorum*, the size distribution of necrotic lesions after 4 days incubation ranged from 0.67 to 8.43 cm, and all genotypes developed a lesion, even the most resistant genotypes, indicating that an infection had successfully occurred in all the plants assayed. Samples were removed from analysis if: the standard deviation of their phenotypic values was ​​greater than 2.0 cm (19 genotypes), the range of their major and minor phenotypic values ​​was greater than 5.0 cm (five genotypes), viral infected or miss-labeled (four genotypes). The remaining 324 genotypes had an approximate normal distribution of phenotypes (Additional file 3: Figure S3) and an average lesion length of 4.65 cm (Additional file 4: Table S1), and were used in for SNP analysis. The genotypes that were the most resistant, including genotypes like EMGOPA 316 [11], PI194639 [8, 9], and NK S19–90 which have been reported to have partial resistance, are shown in Additional file 5: Table S2. The most susceptible, including genotypes like BRSGO Ipameri, BRS RAISSA RES 1/3 NCS, and MSOY 7908 RR which have previously been shown to be very susceptible [10], are listed in Additional file 6: Table S3.

### Genotyping

The HindIII and MseI restriction enzyme double digestions produced a wide peak of fragments in the range of 300–500 bp with little strong banding (Additional file 1: Figure S1F), and therefore this enzyme pair was used for construction of the GBS libraries. The HindIII adaptors were barcoded, allowing all the libraries to be pooled and sequenced on a single lane of an Illumina flow cell, yielding 373 million high-quality, single-stranded readings of 100 nucleotides in length.

**Fig. 1:**
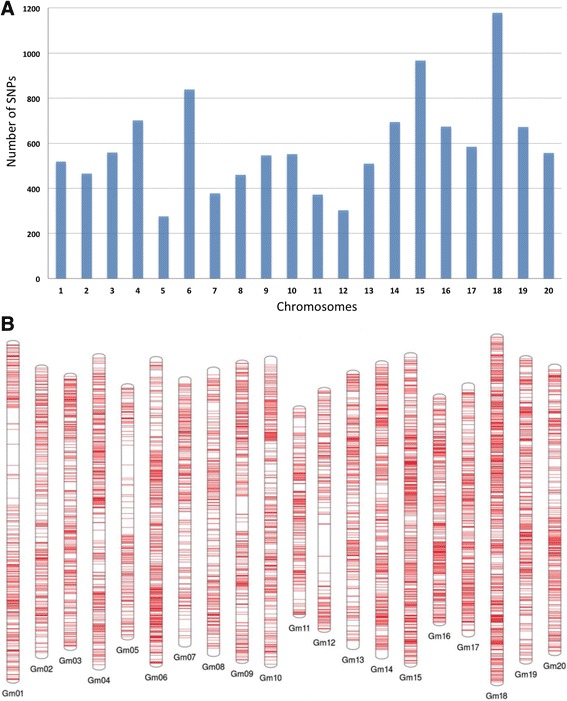
Distribution of the 11,811 SNPs that remained after filtering, and MAF > 0.01, across all 20 soybean chromosomes. **a** Histogram of counts per chromosome. **b** Graphic representation of physical locations of all the SNPs on each chromosome

**Fig. 2:**
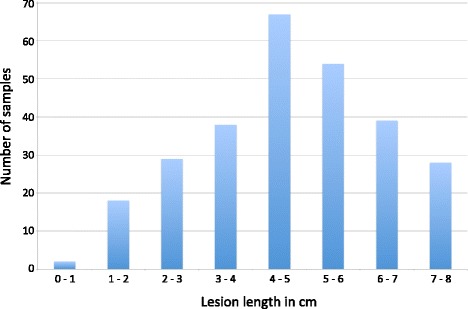
Distribution of phenotypes (lesion length in cm) of the 275 genotypes used in the study. Complete list is provided in Additional file: Table S1

With the TASSEL 5 GBS v2 SNP-calling pipeline, 68,242 SNPs were identified. After filtering for less than 50% missing datas 14,637 SNPs remained. After imputation and removing high heterozygosity, 12,411 SNPs remained. Among them, 11,811 SNPs met the MAF threshold at 0.01, and 6478 SNPs at MAF >0.1. The 11,811 SNPs spread across all chromosomes (Fig. 1), with chromosome 18 containing the most SNPs (1179) and chromosome 5 the fewest (276) SNPs. Filtering of genotypes that had missing data at 75% or more of the SNP sites, or that showed more than 10% heterozygous SNPs, reduced the samples to 275 genotypes for use in association of SNPs to phenotypes. The phenotypes of these 275 genotypes were fairly normally distributed (Fig. 2) and the most resistant (Table 1) and most susceptible (Table 2) are listed.

**Table 1:** Most resistant genotypes (<3.0 cm, 1.5 s.d., 3.0 max range)

Genotype	Score (cm)	Stand Dev	Range cm
FT-1	0.67	0.21	0.60
P1C142–128.686	0.89	0.58	1.70
P1C142–158.243	1.08	1.29	2.70
EMGOPA 316	1.14	0.45	1.50
P1C142–116.940	1.25	0.79	2.00
IAC 100	1.25	1.31	2.70
V-Max RR	1.35	1.08	2.60
FMT05–40.907/1	1.36	0.47	1.30
FT-3	1.46	0.34	1.10
P1C142–102.143	1.60	0.79	1.90
P1C142–123.850	1.60	0.75	2.20
P1C142–127.559	1.65	0.83	2.50
P1C142–126.986	1.67	0.86	2.40
P1C142–112.208	1.73	0.97	2.00
P1C142–170.130	1.80	1.14	2.60
V-Top RR	1.82	0.69	1.90
P1C142–167.428	1.88	0.54	1.20
P1C142–151.788	1.94	0.89	1.80
P1C142–151.674	1.98	0.51	1.40
BMX Turbo RR	2.00	0.73	2.30
P1C142–126.380	2.06	0.36	1.10
L79–1404	2.08	0.79	1.70
P1C142–169.669	2.08	0.74	2.10
L91–8052	2.08	0.87	2.30
P1C142–120.656	2.10	0.85	2.40
P98Y30	2.11	1.29	2.90
P1C142–168.153	2.20	0.88	2.40
P1C142–124.500	2.23	0.45	1.40
FT-37	2.27	1.04	2.90
P1C142–168.971	2.35	1.28	2.80
P1C142–116.766	2.40	0.59	1.40
P1C142–107.220	2.45	0.87	1.90
P1C142–124.193	2.47	0.57	1.10
P1C142–117.050	2.63	0.83	2.50
FT-9	2.78	0.90	2.50
FT-46	2.81	1.03	3.00
P1C142–122.870	2.87	1.14	3.00
NK S19–90	2.98	1.01	2.70

**Table 2:** Most susceptible genotypes (>6.25 cm, <1.5 s.d., 4.0 cm max range)

Genotype	Score (cm)	Stand Dev	Range (cm)
L196	7.68	0.89	2.40
BRSGO Ipameri	7.62	0.69	1.70
PI196157	7.57	0.87	2.70
L25	7.46	0.96	2.80
L379 (T50)	7.41	0.42	1.10
TC12–0-49.220	7.34	0.60	1.60
FT-64	7.33	0.60	1.70
FT-22	7.30	0.37	1.10
L32	7.30	0.35	1.10
TC12–1-50.655	7.29	0.34	1.00
L19	7.29	0.77	2.70
TC12–2-56.681	7.29	0.90	3.00
BR058114 RR	7.28	0.64	1.50
TC12–0-52.243/G022	7.22	0.63	1.80
TMGER15 31,787	7.18	0.64	2.20
BRS RAISSA RES 1/3 NCS	7.18	1.46	4.00
CHAPMAN	7.16	0.82	2.40
P1C142–101.828	7.15	0.49	1.40
MSOY 7908 RR	7.12	0.69	2.20
PI358318A	7.09	0.96	3.40
TMGER15 31,786	7.09	0.55	1.60
TC12–0-52.294	7.05	0.51	1.40
TC12–0-51.186	7.03	1.02	3.00
TC12–0-50.332/G027	7.02	1.04	3.30
TC130–00.011	7.01	0.37	1.10
L263/264 CT	6.89	1.15	3.80
BR05736154	6.84	1.36	3.10
G004501413260 - A	6.77	0.70	1.90
TC12–1-47.590	6.71	0.36	1.20
BSR 101	6.69	1.07	2.50
TC12–2-60.290	6.68	1.22	4.00
PI189861	6.64	1.27	3.60
L671 (T3)	6.59	1.02	2.80
FT-71	6.59	1.11	3.40
TC12–0-46.100	6.56	0.34	1.20
Pickett	6.53	0.88	2.50
L165 (T92)	6.50	0.35	1.10
PI88788	6.49	1.22	3.90
TC12–0-52.024	6.49	0.38	1.00
P1C142–195.052	6.48	0.67	1.60
TC12–0-48.687	6.47	0.44	1.10
TC12–0-52.213	6.43	0.79	2.30
P1C142–185.974	6.41	0.57	1.60
TC12–0-52.294/G026	6.37	0.38	1.20
TC12–0-51.331	6.37	0.92	3.30
P1C142–170.329	6.27	0.43	1.20
P1C142–170.215	6.26	0.49	1.30

### Association analysis using SNP data from the TASSEL 5 GBS v2 SNP-calling pipeline

**Fig. 3:**
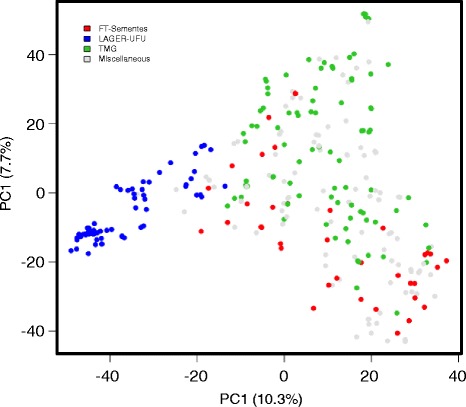
PCA scatter plot showing the two main principal components (PC1 and PC2)

A principal component (PC) analysis was performed with the 11,811 SNPs with a MAF >0.01 and the 275 soybean genotypes to estimate the population structure. Based on the resulting scree plot (Additional file 2: Figure S2), the first three PCs were included in the association analysis. PC1 explained 10.3% of the genetic variation, PC2 explained 7.8%, and PC3 explained 5.5%. The PC analysis scatter plot (Fig. 3) showed that the first and second PCs were composed largely of three subpopulations of genotypes originiaing from different sources. The subpopulation “LAGER-UFU” was more obviously separated while the subpopulations ‘FT-Sementes’ and ‘TMG’ were more close to each other. Soybean genotypes in the ‘miscellaneous’ group were collected from different sources, including many that were selected based on the literature.

**Fig. 4:**
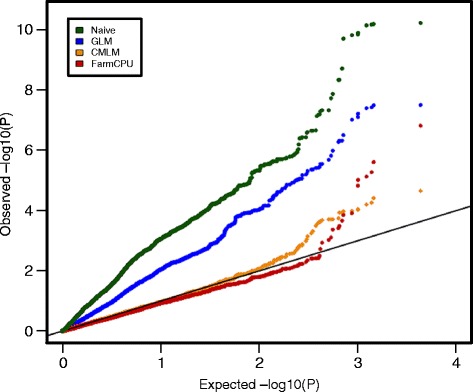
Quantile-quantile (QQ) plots comparing different GWAS models using data from the in-house SNP calling pipeline. GLM: generalized linear model, CMLM: compressed mixed linear model, FARMCPU: Fixed and random model Circuitous Probability Unification. Note: when plotted, the MLM overlapped with the CMLM points, and therefore, only the CMLM plot is shown

Four different models were implemented and compared in the marker-trait association tests. Q-Q plots were generated to visualize the effectiveness of controlling population structure and familial relatedness (Fig. 4). The expected distribution of *p*-values is a uniform [0,1] distribution under the assumption that no associations exist between SNP markers and the trait. The naïve model and the GLM model resulted in 38.7% and 23.1% of SNPs with *p*-values <0.05, respectively and it can be seen from the Q-Q plot that both models produced a large proportion of *p*-values that deviated from the expected distribution, which indicated an excess of false positives. Compared to the naïve model and the GLM, the CMLM and the FarmCPU resulted in 3.8% and 4.9% of SNPs with *p*-values <0.05, indicating the inflation of *p*-values was reduced and supported the appropriateness of including population structure and kinship matrix in the model. In addition, FarmCPU yielded most of the SNPs fitting the expected distribution of *p*-values with a few SNPs exhibiting high significance (higher significance than CMLM).

**Fig. 5:**
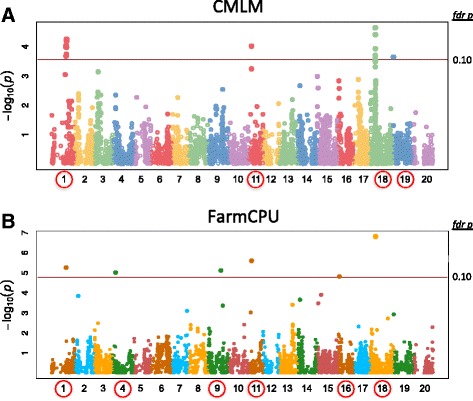
Manhattan plots of the association of the SNPs with the phenotype of defense to *S. sclerotiorum* generated by (**a**) GAPIT/CMLM or (**b**) FarmCPU. The chromosomal SNPs are differentiated by various colors, and the FDR-corrected *p*-value =0.1 is delineated with a horizontal orange line. The left axis is the raw *p*-value

Because the CMLM and FarmCPU both seemed appropriate for analyzing the association between the soybean SNPs and SSR resistance, the association results from CMLM (conducted in the R package GAPIT) and FarmCPU (conducted in the R package FarmCPU) were compared. Setting the FDR-corrected *p*-value cutoff at 0.05, six SNPs (on chromosomes 1, 4, 9, 11, 16, and 18) were detected as significant by FarmCPU, with each significant SNP explaining approximately 2% to 3.2% of the total phenotypic variation (Table 3). The CMLM analysis did not identify any SNPs as significantly associated at the 0.05 FDR-corrected *p*-value cutoff. But loosening the strict threshold to an FDR-corrected *p*-value <0.1, a total of 19 SNPs (on chromosomes 1, 11, 18 and 19) showed significant associations with CMLM, where each SNP explained 3.2% to 4.4% of the total phenotypic variation (Table 3). No additional SNPs were significant with FarmCPU at the <0.1 cutoff. The three most significant SNPs detected by FarmCPU (S1_36,045,483, S11_6,493,121 and S18_14,327,556) were also deemed significant by CMLM, as shown by the Manhattan plot (Fig. 5 and Table 3). However, the SNPs on chromosomes 4, 9 and 16 that were identified with FarmCPU as significant, were not significant by CMLM, although weak signals could be seen at those sites on the CMLM-produced Manhattan plot (Fig. 5). A single SNP on chromosome 19 determined by CMLM did not pass the significance threshold with the FarmCPU, but there was a weak peak on the FarmCPU-produced Manhattan plot (Fig. 5). The Manhattan plots generated by the two models look different for the significant loci, as the LD associated with the SNPs affected whether there would be a string of hits (on the CMLM plot) or a single spot (on the FarmCPU plot) as FarmCPU removed all the SNPs in LD, and these SNPs in LD were kept by the CMLM analysis (Fig. 5).

**Table 3:** SNPs associated with the defense response at fdr p-value <0.10

GAPIT/CMLM								
SNP	Chromo	Position	Alleles	MAF	p-value	FDR p-value	R2	Allelic Effect
S18_14,327,556*	18	14,327,556	T/C	0.491349481	2.23E-05	0.0751889	4.41%	0.81844
S18_14,327,607	18	14,327,607	A/G	0.493079585	3.86E-05	0.0751889	4.15%	−0.79800
S1_36,783,951	1	36,783,951	G/A	0.264705882	5.50E-05	0.0751889	3.98%	0.65452
S1_36,497,505	1	36,497,505	A/G	0.269896194	6.33E-05	0.0751889	3.91%	−0.63779
S1_35,474,053	1	35,474,053	C/T	0.261245675	9.03E-05	0.0751889	3.75%	−0.62749
S11_6,493,121*	11	6,493,121	T/C	0.228373702	9.48E-05	0.0751889	3.72%	−0.58271
S1_35,045,463	1	35,045,463	C/T	0.26816609	1.03E-04	0.0751889	3.69%	−0.62200
S1_36,006,734	1	36,006,734	A/G	0.271626298	1.06E-04	0.0751889	3.67%	−0.60778
S18_14,517,407	18	14,517,407	C/A	0.489619377	1.17E-04	0.0751889	3.62%	0.74685
S18_14,517,362	18	14,517,362	C/T	0.489619377	1.17E-04	0.0751889	3.62%	−0.74685
S1_36,045,483*	1	36,045,483	G/A	0.262975779	1.81E-04	0.0878468	3.42%	0.58989
S18_14,282,760	18	14,282,760	A/G	0.496539792	1.97E-04	0.0878468	3.38%	0.72465
S18_14,282,812	18	14,282,812	A/G	0.496539792	1.97E-04	0.0878468	3.38%	0.72465
S18_14,282,806	18	14,282,806	T/C	0.496539792	1.97E-04	0.0878468	3.38%	−0.72465
S1_35,152,187	1	35,152,187	T/G	0.271626298	2.09E-04	0.0878468	3.35%	0.59070
S19_1,289,850	19	1,289,850	A/G	0.155709343	2.25E-04	0.0878468	3.32%	−0.61268
S18_14,324,249	18	14,324,249	G/T	0.496539792	2.46E-04	0.0878468	3.27%	0.71140
S18_14,324,245	18	14,324,245	T/C	0.496539792	2.46E-04	0.0878468	3.27%	−0.71140
S18_14,334,212	18	14,334,212	T/C	0.5	2.86E-04	0.0968226	3.20%	−0.69934
FarmCPU								
SNP	Chromo	Position	Alleles	MAF	p-value	FDR p-val	R2	Allelic Effect
S18_14,327,556*	18	14,327,556	T/C	0.491349481	1.54E-07	0.0009911	3.26%	0.64476
S11_6,493,121*	11	6,493,121	T/C	0.228373702	2.48E-06	0.0079806	2.44%	−0.41685
S1_36,045,483*	1	36,045,483	G/A	0.262975779	5.38E-06	0.0115419	2.29%	0.40215
S9_32,113,409	9	32,113,409	C/T	0.335640138	7.51E-06	0.0120836	2.25%	0.34200
S4_7,210,961	4	7,210,961	C/G	0.249134948	9.64E-06	0.0124086	2.27%	0.38084
S16_3,111,366	16	3,111,366	G/A	0.14532872	1.50E-05	0.0160900	2.09%	−0.46977

* SPNs marked were significant by both GAPIT/CMLM and FarmCPU

### LD and candidate genes analysis of three major loci

**Fig. 6:**
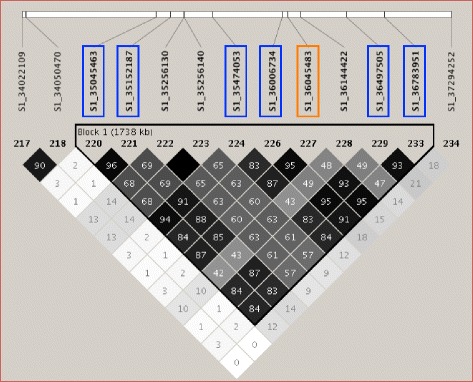
LD plot of the genomic regions on chromosome 1 harboring the significant SNP in common between CMLM and FarmCPU (*orange box*) and the significant SNPs identified by CMLM (*lavender boxes*). Numbers in squares indicate 100-fold r^2^ values of each pair of SNPs. The intensity of gray represents the level of r^2^. The bars above LD plots represent physical positions of SNPs. Black triangles that outline parts of LD plots indicate the defined LD blocks

**Fig. 7:**
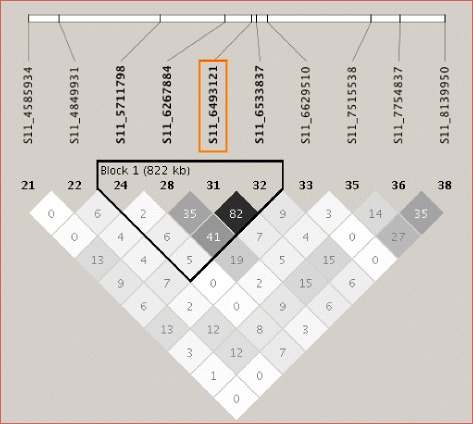
LD plot of the genomic regions on chromosome 11 harboring the significant SNP in common between CMLM and FarmCPU (*orange box*). Numbers in squares indicate 100-fold r^2^ values of each pair of SNPs. The intensity of gray represents the level of r^2^. The bars above LD plots represent physical positions of SNPs. Black triangles that outline parts of LD plots indicate the defined LD blocks

**Fig. 8:**
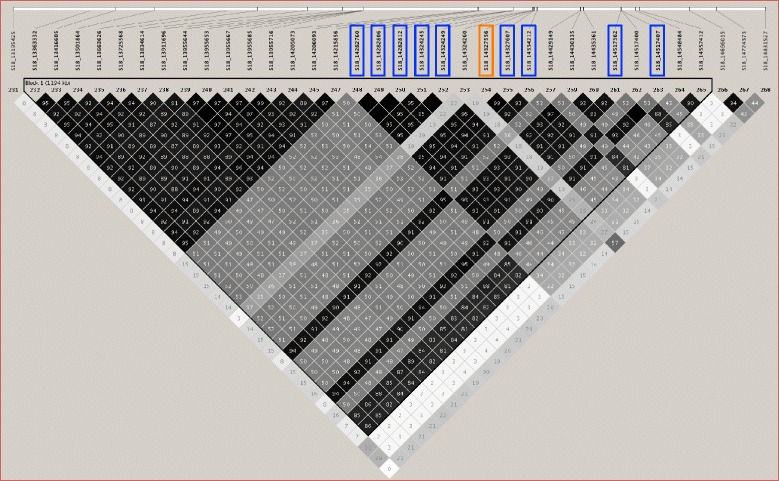
LD plot of the genomic regions on chromosome 18 harboring the significant SNP in common between CMLM and FarmCPU (*orange box*) and the significant SNPs identified by CMLM (*lavender boxes*). Numbers in squares indicate 100-fold r^2^ values of each pair of SNPs. The intensity of gray represents the level of r^2^. The bars above LD plots represent physical positions of SNPs. Black triangles that outline parts of LD plots indicate the defined LD blocks

Because both CMLM and FarmCPU analyses identified significant SNPs on chromosome 1, 11 and 18, these three loci were considered to be the most significant of the study, and further analyses were therefore performed on them. LD plots were generated for these three loci to examine the local LD blocks. Seven significant SNPs on chromosome 1 were located within a LD block of 1738 kb from 35 to 36.8 Mb, and all SNPs in this LD block showed moderate to high r^2^ values from 0.4 to 1 (Fig. 6), indicating that these seven SNPs were highly associated, and therefore might have the same causal site(s) that contributed to enhanced resistance. The significant SNP on chromosome 11 lied within an LD block of 822 kb (Fig. 7). However, since the LD block was determined by D’ values, the SNP at 5711798 bp showed fairly low LD with the other three SNPs in the block considering r^2^ values (r^2^ < 0.1). Three SNPs from 6.2 Mb to 6.5 Mb showed high r^2^ values with each other, suggesting the r^2^-based LD block here was about 0.3 Mb (Fig. 7). Another large (1194 kb) LD block was located around the most significant SNP on chromosome 18, from 13.3 Mb to 14.5 Mb. By the CMLM analysis, this block contained ten significant SNPs that exhibited very high pairwise r^2^ values >0.9 (Fig. 8).

The soybean reference genome [46] was queried to identify the genes that predicted to be within the LD blocks. The 1.7 Mb LD block around the SNP peak on chromosome 1 contained 36 genes, the 0.3 Mb LD block on chromosome 11 contained 35 genes, and the 1.1 Mb LD block on chromosome 18 contained 54 genes (Additional file 7: Table S4).

**Fig. 9:**
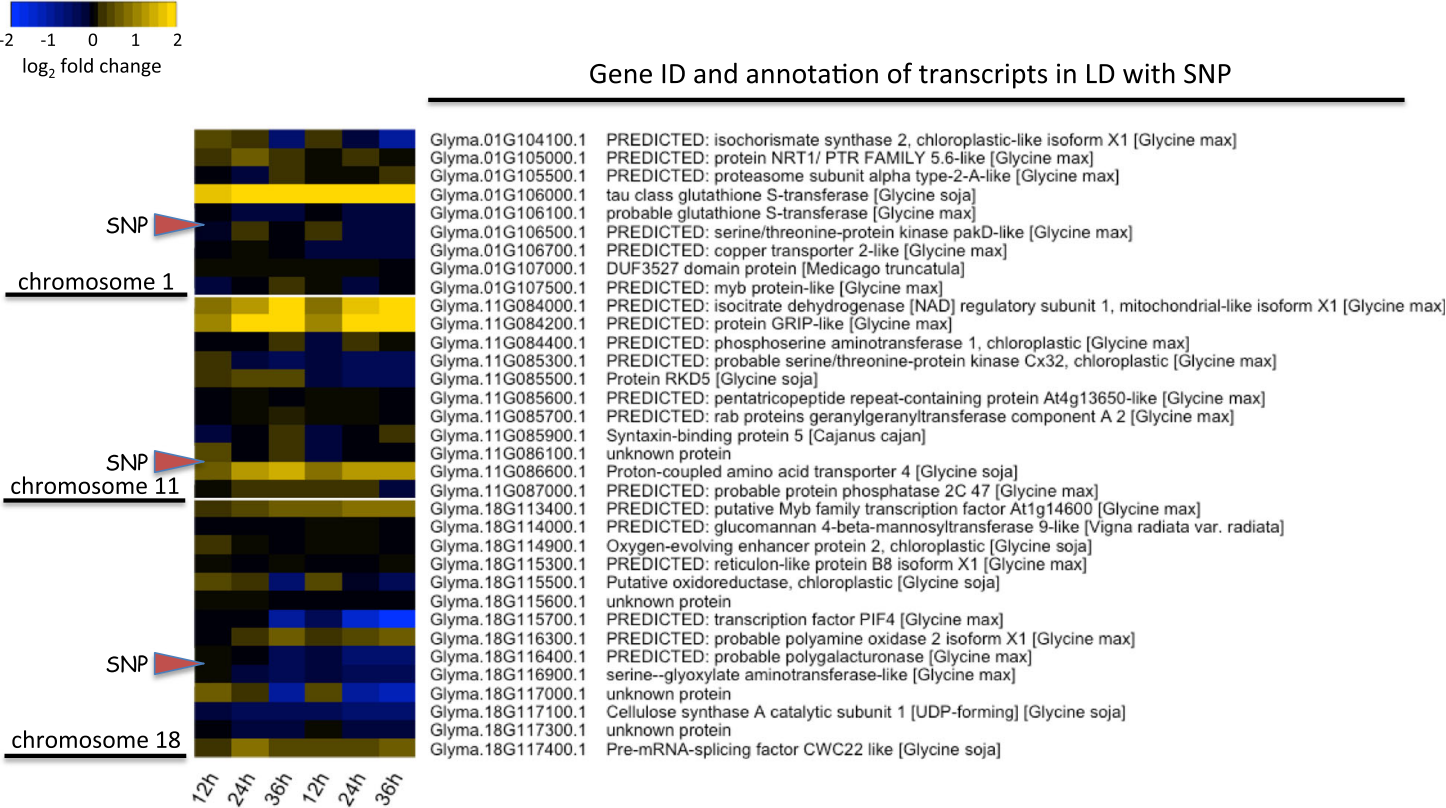
Heat map of differential expression of genes within region of significant SNPs on chromosomes 1, 11, and 18. The complete list of genes in LD with these SNPs is in Additional file: Table S4, the genes shown here are only those for which microarray expression data [12] from *S. sclerotiorum* infected soybean tissue was available. Expression values are the log_2_ ratios of inoculated genotypes versus mock inoculated, at 12, 24, and 36 h post inoculation

The number of genes within the LD blocks on chromosomes 1, 11, and 18 totalled 125. The translations of the putative coding sequences of these genes were aligned by blastx to the nr database of NCBI [47] to identify possible gene function (Additional file 7: Table S4). The genes with the LD blocks were also checked for their expression pattern in a microarray experiment involving soybean transcriptome response to *S. sclerotiorum* infection [12]. Although most of the genes showed moderate to no expression change in response to infection, 34 of the 125 genes were identified as being differentially expressed (inoculated vs mock) during the first 36 h post inoculation (hpi) (Fig. 9). There were sixteen genes with significant differential expression between infected samples and mock treated samples in at least one time point, making them more promising candidates for genes potentionally involved in defense efforts. Several of the genes in the microarray study (Fig. 9) showed a much stronger expression during *S. sclerotiorum* infection than the others. The gene with the strongest differential expression was Glyma.01G106000, that encodes a tau class glutathione S-transferase; it had the strongest expression differences in both genotypes used in that sudy, at 12, 24, and 36 hpi, and is about 189.0 kb away from the peak SNP on chromosome 1, S1_36,045,483. Three genes on chromosome 11, Glyma.11G084000, Glyma.11G084200, and Glyma.11G086600, that are in LD with the peak SNP S11_6,493,121, also showed fairly strong induction of expression after infection compared to other genes. Other genes that were part of the microarray study did not change any more than 2 fold in response to inoculation (Fig. 9).

## Discussion

The study was successful in identifying SNPs significantly associated with soybean defense against SSR, a disease that is increasing in importance worldwide, and whose resistance QTL are very challenging to map due to the high phenotypic variability of the disease, and the minor contribution from each QTL. The use of GWAS with thousands of SNP markers, improves the ability to identify QTL with higher statistical significance, and several other research groups have already applied GWAS to identify loci associated with SSR responses [28, 29, 31].

### GWAS succeeded in identifying new QTL associated with SSR

In this presented GWAS of soybean resistance to SSR, six QTL were identified using the FarmCPU computation, and four with the CMLM computation, with an overlap of three QTL identified by SNPs: S1_36,045,483, S11_6,493,121, and S18_14,327,556. The study did not have good overlap with the QTL identified in the other recent GWAS studies, except the possible overlap of our QTL on chromosome 1 near 35.5 Mb, to the QTL near 27.7 Mb (position updated to the same version of the soybean reference genome sequence) of Bastein et al. (2014). The QTL on chromosome 19 was only identified as significant by CMLM, but it might be the same identified by Vuong et al. (2008) where a QTL for SSR resistance in PI194639 was bordered by Satt495 (Chr19:650,674) and Satt388 (Chr19:4,212,645) [9, 48]. This lack of overlapping QTL identification between different experiments, might reflect the subtle effects of each QTL for SSR resistance or the high variability in phenotyping this disease. The lack of QTL overlap could also be due to the use of different soybean genotypes, the use of different pathogen isolates that were collected on different continents, or the use of different inoculation methods or treatments. Among the other GWAS, two of the studies inoculated flowers and measured disease progression down the stem seven days later [28, 29], and the other study treated soybeans with oxalic acid released by *S. sclerotinia*, at 40 mM, and measured pigment induction in response to this treatment 48 h later [31]. In the present study, young seedlings were inoculated on freshly cut stems. Therefore, it is not too surprising that the QTL identified in each study could differ.

### How CMLM and FarmCPU compare

The model chosen to conduct GWAS should be carefully decided based on species and traits being studied. For this study of soybean, it involved a plant that is self-pollinated and with high LD across the genome, and resistance to SSR is underlied by multiple genes with small effects. The analysis tested two different methods, CMLM and FarmCPU, for marker-trait association. The Q-Q plot showed both CMLM and FarmCPU controlled inflated *p*-values due to population structure better than the other more naïve models. The FarmCPU method identified more significant loci and with higher levels of significance for the most significant SNPs.

The mixed linear model has been widely used in GWAS studies on soybean and in a variety of other crops as it was shown to largely reduce the spurious associations resulting from both population structure and unequal individual relatedness [49–53]. Based on the MLM, the CMLM implemented in the GAPIT package to deal with large and computational challenging datasets by clustering individuals into groups in the kinship matrix [32, 54]. Although popular for GWAS, in some cases the CMLM can be insufficient due to the confounding between the population structure, kinship, and different testing SNPs, which could potentially lead to compromise of true postives. It was reported that the recently developed method FarmCPU mitigated this problem and led to both increased statistical power and reduced false positives [33]. FarmCPU implements a fixed effect model that contains the testing markers and multiple associated markers as covariates, and a random model that contains the kinship matrix. These steps are performed separately but optimize each other iteratively. So compared to CMLM in which the kinship matrix remains constant for all the markers, FarmCPU adjusts its kinship based on the testing markers and covariates in the fixed effect model. Moreover, since the CMLM only tests one marker at a time, other associated loci nearby or elsewhere in the genome will sometimes disrupt with the test and resulted in false positives or false negatives, especially when the effects of the other loci are large [55]. Therefore, FarmCPU puts selected associated markers as covariates and tests multiple markers simultaneously, thus improving control of both false positives and false negatives [33]. This could be illustrated by comparing the Manhattan plots (Fig. 5) where “spikes” consisting of multiple SNPs in LD are present with CMLM, but only single SNPs, representing the most significant association, appear with FarmCPU. However, FarmCPU has a weakness in that removing the significant SNPs in LD with the peak SNPs, reduces information, and these SNPs in LD help to confirm the existence of an truly associated locus. Considering upsides and downsides, it is beneficial to implement different models to do association studies to increase confidence in the overlapping loci, and to also increase the possibility of identifying unique novel loci.

### Examining LD and genes around the significant SNPs

Soybean has a relatively high LD genome-wide, and LD increases extensively in the pericentromere regions [28, 56, 57]. In our study, seven and ten significant SNPs on chromosome 1 and 18 were found to be in megabase-level LD blocks in pericentromeric regions, where genes are sparsely distributed according to the soybean reference genome. The size of the LD blocks could also be affected by the density of the SNP markers. For example, the closest marker next to the LD block from 35,045,463 bp to 36,783,951 bp on chromosome 1 was 1 Mb away, at 34050470 bp, so there were no SNPs indicating where the LD block terminated in this 1 Mb region. A higher density SNP map would help refine the large LD, and perhaps narrow the region of interest.

Looking at the genes that are located in LD with the SNPs, and combining soybean gene expression data after *S. sclerotiorum* infection, can increase the confidence in identifying candidate SSR defense-associated genes. Two of the more promising genes in LD with the QTL identified on chromosome 1 based on gene expression were Glyma.01G104100 and Glyma.01G106000. Likewise, the most promising genes on chromosome 11 were three that showed the strongest induction in response to *S. sclerotiorum* infection: Glyma.11G084000, Glyma.11G084200, and Glyma.11G086600. The genes in LD with the significant SNPs on chromosome 18, were weakly differentially expressed, with the strongest ones, like Glyma.18G113400 and Glyma.18G117400, showing about a 2-fold induction.

Of the genes that are in LD with the significant SNPs on chromosome 1, and whose expression were induced by *S. sclerotiorum* infection, Glyma.01G106000 was one of the most interesting as it was strongly induced over all time points and samples, and also significantly induced after *Pseudomonas syringae* infection on soybean [58]. Glyma.01G106000 encodes a tau class glutathione S- transferase (GST) protein, which may be involved in the process of xenobiotic detoxification, reduction of organic hydroperoxides, or oxidative protection [59, 60]—all common needs during plant-pathogen interactions. Overexpression of a rice tau-class GST increased the tolerance to salinity and oxidative stress in *Arabidopsis thaliana* and tobacco, which may be due to the lower accumulation of reactive oxygen species [61, 62]. Another interesting defense-related gene near the QTL on chromosome 1 is Glyma.01G104100 which encodes an isochorismate synthase, the key enzyme in the synthesis of salicylic acid. This gene was not strongly induced by infection, and was actually reducing over time (Fig. 9). However, salicylic acid is a very important regulatory signal in plant microbe interactions [63, 64], especially for the host-related cell-death pathways [65, 58]. Inducing cell death is believed to be a beneficial strategy that necrotrophic pathogens utilize, and *S. sclerotiorum* has been shown to manipulate host cell death to its advantage [14]. That salicylic acid is known to induce cell death, and that *S. sclerotiorum* benefits from enhanced cell death, expression of this isochorismate synthase might enhance susceptibility. Interestingly, the allele present in both of the genotypes of the microarray study is predicted to have a negative effect on defense according to the GWAS results.

Looking at the genes within LD of the significant SNPs on chromosome 11, most of the genes seem to be related to primary metabolism. Although not directly associated with defense, primary metabolism may also affect the outcome of plant pathogen interactions, so the involvement of one of these genes on defense cannot be dismissed. Gene Glyma.11G084200 was strongly induced and putatively encodes a GRIP-like protein. GRIP proteins was shown to target the golgi [66], but their potential function in plant-pathogen interactions is unknown.

The genes within LD of the significant SNPs on chromosome 18 were more weakly differentially expressed than some of those genes discussed on chromosome 1 and 11. Two genes are associated with cell wall modification, Glyma.18G116400 (a probable polygalacturonase) and Glyma.18G117100 (a cellulose synthase). Although their expression is not significantly affected by *S. sclerotiorum* infection, plant cell wall degrading enzymes play an important role in SSR disease [17]. Another gene of interest within this region on chromosome 18 is Glyma.18G113400, which encodes a putative Myb transcription factor, increased in expression after *S. sclerotiorum* infection along the time course, from 12 to 36 h (Fig. 9). It was shown that Myb transcription factors can be involved in various biological processes in plants, including response to biotic stresses [67, 59].

## Conclusions

Using different genetic materials and inoculation methods than the other recent SSR GWAS reports, we succeeded to identify new QTL associated with soybean resistance to SSR disease caused by *S. sclerotiorum*. The study identified four or six SSR resistance QTL, depending on GWAS computational analysis performed. Three of the QTL (on chromosomes 1, 11, and 18) were detected by both methods, giving more confidence that they are signicantly associated with soybean defense to SSR disease. Looking at the genes within the LD blocks of the significant QTL allowed for prediction of candidate defense-associated genes responsible for the enhanced resistance. One of the most interesting genes in these LD blocks was Glyma.01G104100, which encodes an isochorismate synthase, and could play a role in regulating host cell death pathways. Further experimentation will be needed to verify the relevance of the identified SNPs and putative gene functions in the soybean- *S. sclerotiorum* interaction.

## Additional files

Additional file 1: Figure S1. Testing Restriction_Enzymes. (DOCX 508 kb)

Additional file 2: Figure S2. Scree plot of eigen values to determine number of principle components for the GWAS models. (PDF 8 kb)

Additional file 3: Figure S3. Distribution of 324 phenotypes. (PDF 99 kb)

Additional file 4: Table S1. Phenotypes of all 324 genotypes used. (XLSX 31 kb)

Additional file 5: Table S2. Top resistant genotypes prior to filtering based on SNPs. (DOCX 87 kb)

Additional file 6: Table S3. Top susceptible genotypes prior to filtering based on SNPs. (DOCX 86 kb)

Additional file 7: Table S4. All genes in the LD blocks containing the significant SNPs on chromosome 1, 11 and 18. (XLSX 45 kb)

## Abbreviations

FarmCPU: Fixed and random model Circulating Probability Unification
GAPIT: Genome Association & Prediction Integrated Tool
GBS: Genotype by Sequencing
GWAS: Genome Wide Association Study
LD: Linkage Disequilibrium
QTL: Quantitative Trait Locus/Loci
