# Supplementary material for: Genome-wide association mapping of resistance to a Brazilian isolate of Sclerotinia sclerotiorum in soybean genotypes mostly from Brazil
Source: BMC Genomics. 2017 Nov 7;18:849. doi: 10.1186/s12864-017-4160-1 (PMC5674791; doi:10.1186/s12864-017-4160-1)
Supplement: Supplementary file 1 — Testing Restriction_Enzymes. (DOCX 508 kb) [file 12864_2017_4160_MOESM1_ESM.docx]

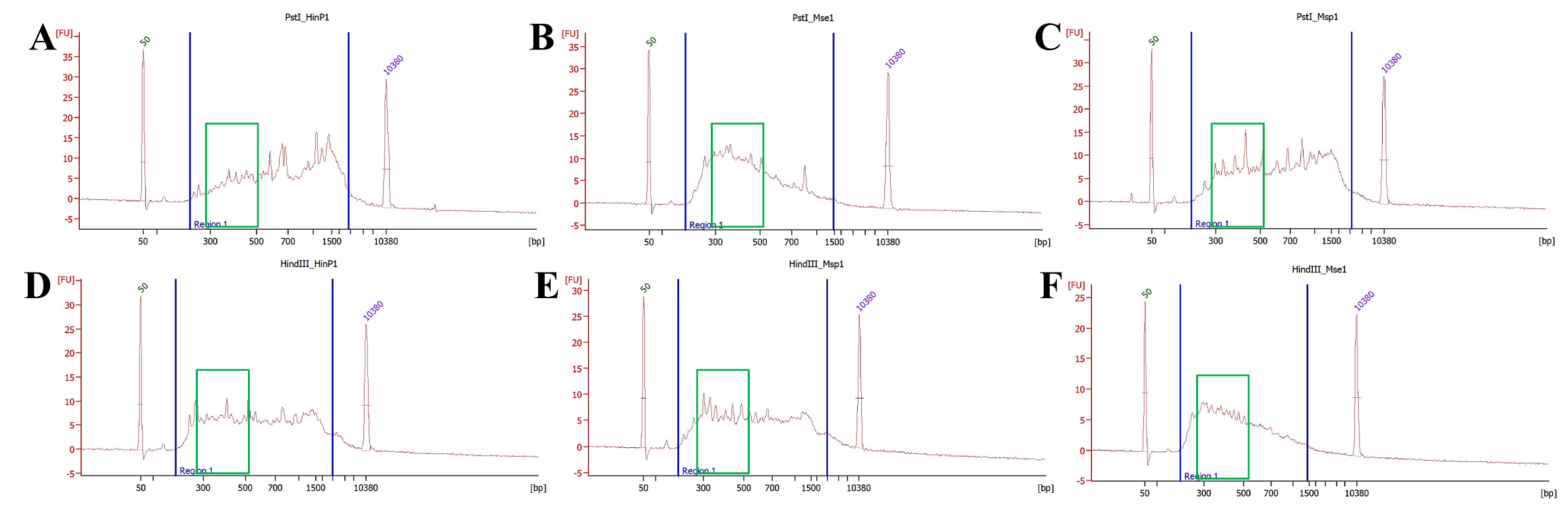
**Additional file 1: Figure S1**. Testing Restriction_Enzymes on the Bioanalyzer 2100. **A.** PstI / HinP1I; **B.** PstI / Mse1; **C.** PstI / MspI; **D.** HindIII / HinP1I; **E.** HindIII / Msp1I; **F.** HindIII / MseI. The green rectangles indicate desired range of fragment sizes (300-500 bp) for the GBS library construction. FU: fluorescence units.
