# Supplementary figures and images for: Genome-wide association mapping of resistance to a Brazilian isolate of Sclerotinia sclerotiorum in soybean genotypes mostly from Brazil
Source: BMC Genomics. 2017 Nov 7;18:849. doi: 10.1186/s12864-017-4160-1 (PMC5674791; doi:10.1186/s12864-017-4160-1)

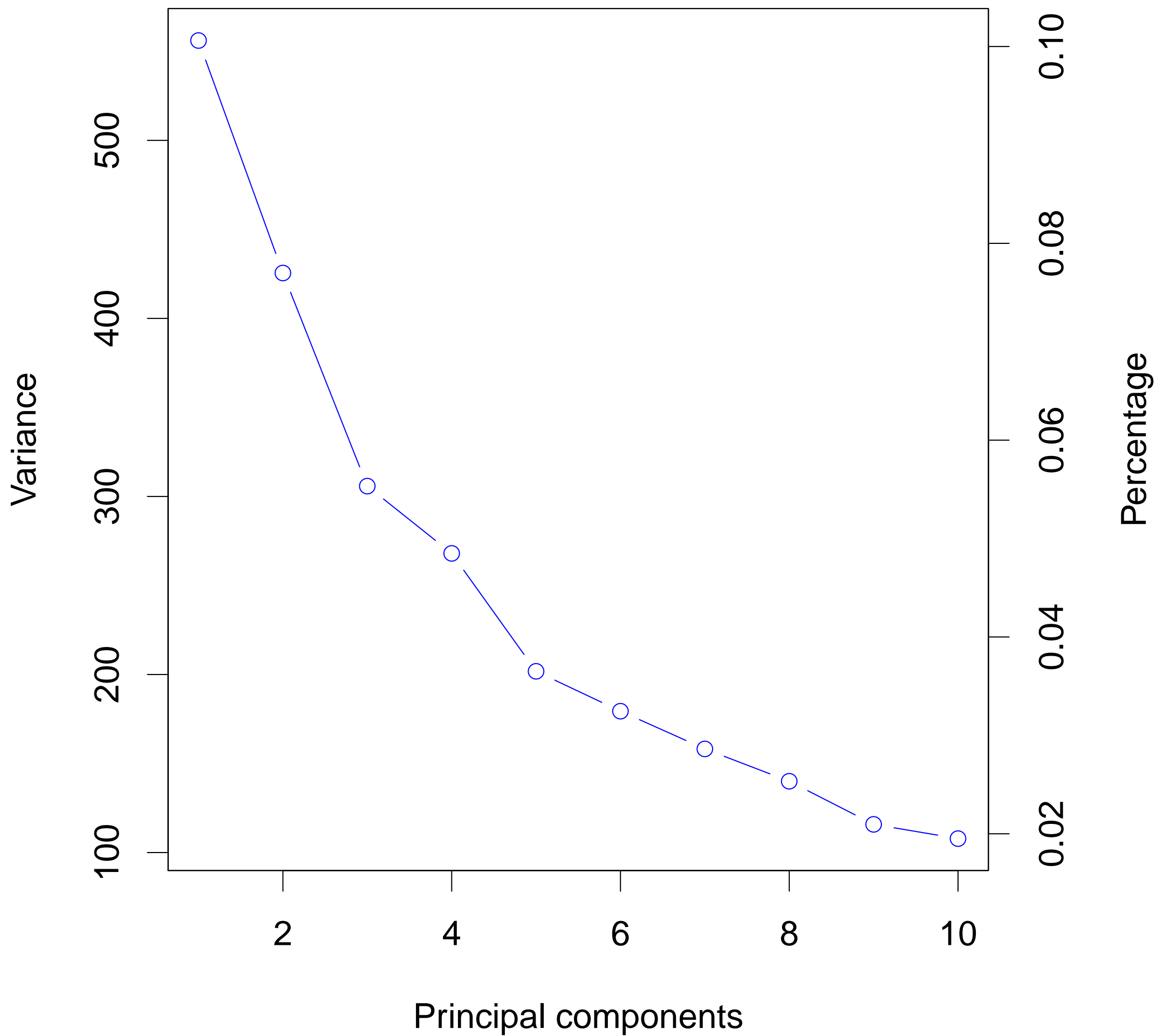

Supplement: Supplementary file 2 — Scree plot of eigen values to determine number of principle components for the GWAS models. (PDF 8 kb) [file 12864_2017_4160_MOESM2_ESM.pdf]

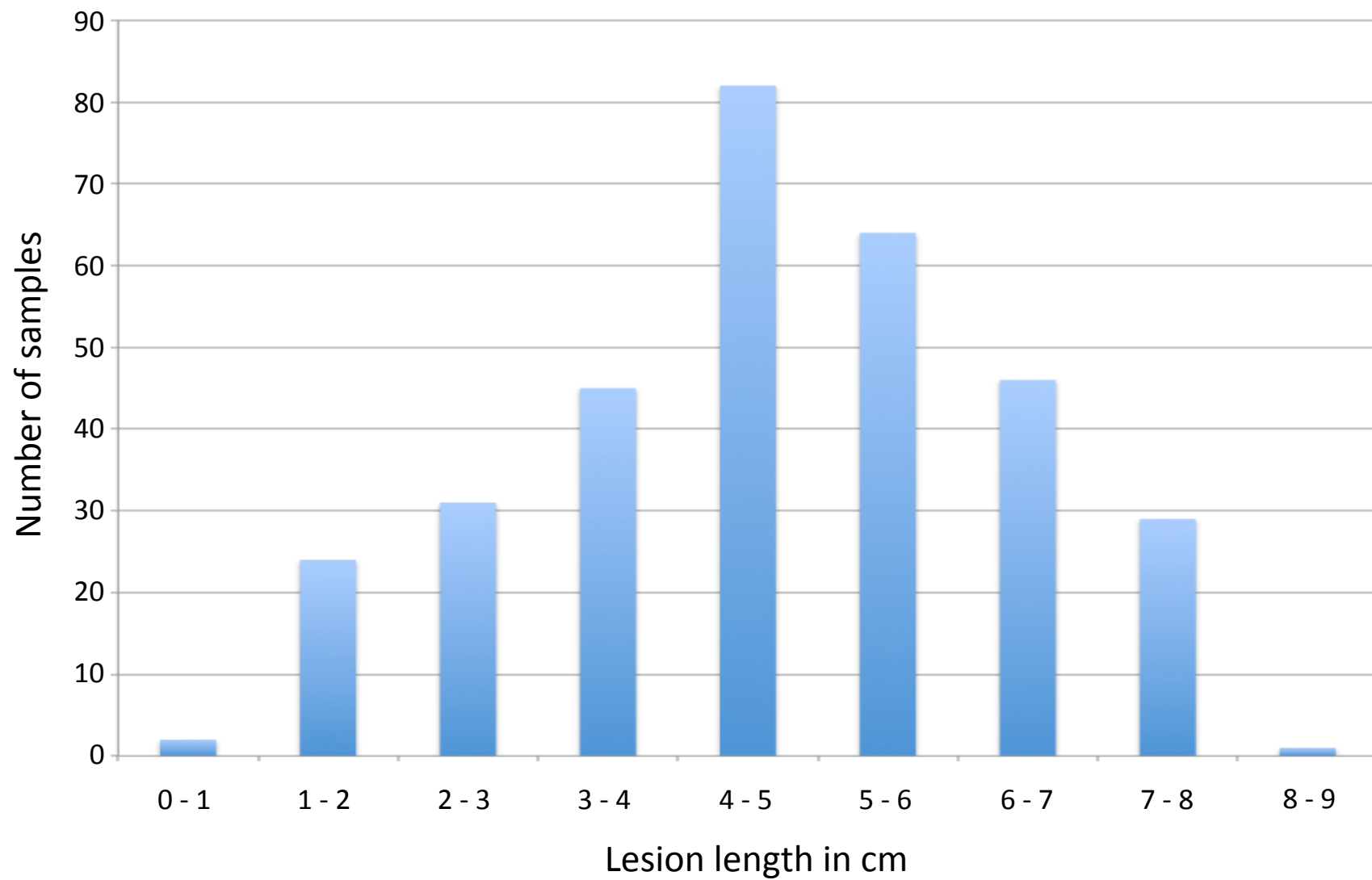

Supplement: Supplementary file 3 — Distribution of 324 phenotypes. (PDF 99 kb) [file 12864_2017_4160_MOESM3_ESM.pdf]
