# Supplementary material for: Genome-wide association mapping of resistance to a Brazilian isolate of Sclerotinia sclerotiorum in soybean genotypes mostly from Brazil
Source: BMC Genomics. 2017 Nov 7;18:849. doi: 10.1186/s12864-017-4160-1 (PMC5674791; doi:10.1186/s12864-017-4160-1)
Supplement: Supplementary file 5 — Top resistant genotypes prior to filtering based on SNPs. (DOCX 87 kb) [file 12864_2017_4160_MOESM5_ESM.docx]

| **Table S2. Most resistant genotypes (<3.0cm, 1.5 s.d., max 3.0 range)** | | | |
| --- | --- | --- | --- |
| **Genotype** | **Score (cm)** | **Stand Dev** | **Range (cm)** |
| FT-1 | 0.67 | 0.21 | 0.60 |
| P1C142-128.686 | 0.89 | 0.58 | 1.70 |
| P1C142-158.243 | 1.08 | 1.29 | 2.70 |
| EMGOPA 316 | 1.14 | 0.45 | 1.50 |
| P1C142-116.940 | 1.25 | 0.79 | 2.00 |
| IAC 100 | 1.25 | 1.31 | 2.70 |
| V-Max RR | 1.35 | 1.08 | 2.60 |
| FMT05-40.907/1 | 1.36 | 0.47 | 1.30 |
| FT-3 | 1.46 | 0.34 | 1.10 |
| P1C142-156.243 | 1.56 | 0.98 | 2.40 |
| P1C142-123.850 | 1.60 | 0.75 | 2.20 |
| P1C142-102.143 | 1.60 | 0.79 | 1.90 |
| P1C142-127.559 | 1.65 | 0.83 | 2.50 |
| P1C142-126.986 | 1.67 | 0.86 | 2.40 |
| P1C142-169.073 | 1.71 | 0.79 | 2.50 |
| P1C142-112.208 | 1.73 | 0.97 | 2.00 |
| P1C142-168.243 | 1.78 | 0.55 | 1.50 |
| P1C142-170.130 | 1.80 | 1.14 | 2.60 |
| PI194639 | 1.81 | 0.38 | 1.00 |
| V-Top RR | 1.82 | 0.69 | 1.90 |
| P1C142-167.428 | 1.88 | 0.54 | 1.20 |
| P1C142-151.788 | 1.94 | 0.89 | 1.80 |
| P1C142-120.884 | 1.97 | 0.58 | 1.40 |
| P1C142-151.674 | 1.98 | 0.51 | 1.40 |
| P1C142-119.350 | 1.99 | 1.03 | 2.60 |
| BMX Turbo RR | 2.00 | 0.73 | 2.30 |
| P1C142-110.417 | 2.02 | 0.96 | 2.40 |
| P1C142-126.380 | 2.06 | 0.36 | 1.10 |
| L79-1404 | 2.08 | 0.79 | 1.70 |
| P1C142-169.669 | 2.08 | 0.74 | 2.10 |
| L91-8052 | 2.08 | 0.87 | 2.30 |
| P1C142-120.656 | 2.10 | 0.85 | 2.40 |
| P98Y30 | 2.11 | 1.29 | 2.90 |
| P1C142-168.153 | 2.20 | 0.88 | 2.40 |
| P1C142-124.500 | 2.23 | 0.45 | 1.40 |
| FT-37 | 2.27 | 1.04 | 2.90 |
| P1C142-168.971 | 2.35 | 1.28 | 2.80 |
| P1C142-116.766 | 2.40 | 0.59 | 1.40 |
| P1C142-107.220 | 2.45 | 0.87 | 1.90 |
| P1C142-124.193 | 2.47 | 0.57 | 1.10 |
| P1C142-117.050 | 2.63 | 0.83 | 2.50 |
| FT-9 | 2.78 | 0.90 | 2.50 |
| FT-46 | 2.81 | 1.03 | 3.00 |
| P1C142-122.870 | 2.87 | 1.14 | 3.00 |
| NK S19-90 | 2.98 | 1.01 | 2.70 |
