# Supplementary material for: Genome-wide association mapping of resistance to a Brazilian isolate of Sclerotinia sclerotiorum in soybean genotypes mostly from Brazil
Source: BMC Genomics. 2017 Nov 7;18:849. doi: 10.1186/s12864-017-4160-1 (PMC5674791; doi:10.1186/s12864-017-4160-1)
Supplement: Supplementary file 6 — Top susceptible genotypes prior to filtering based on SNPs. (DOCX 86 kb) [file 12864_2017_4160_MOESM6_ESM.docx]

| Table S3. Most susceptible genotypes (>6.25 cm, <1.5 s.d., <4.0 cm range) | | | |
| --- | --- | --- | --- |
| Genotype | Score (cm) | Stand Dev | Range (cm) |
| L90 (CT) | 8.43 | 0.59 | 1.40 |
| L196 | 7.68 | 0.89 | 2.40 |
| BRSGO Ipameri | 7.62 | 0.69 | 1.70 |
| PI196157 | 7.57 | 0.87 | 2.70 |
| L25 | 7.46 | 0.96 | 2.80 |
| L379 (T50) | 7.41 | 0.42 | 1.10 |
| ANTA 82 RR | 7.36 | 0.86 | 2.40 |
| TC12-0-49.220 | 7.34 | 0.60 | 1.60 |
| FT-64 | 7.33 | 0.60 | 1.70 |
| FT-22 | 7.30 | 0.37 | 1.10 |
| L32 | 7.30 | 0.35 | 1.10 |
| TC12-1-50.655 | 7.29 | 0.34 | 1.00 |
| L19 | 7.29 | 0.77 | 2.70 |
| TC12-2-56.681 | 7.29 | 0.90 | 3.00 |
| BR058114 RR | 7.28 | 0.64 | 1.50 |
| TC12-0-52.243/G022 | 7.22 | 0.63 | 1.80 |
| TMGER15 31787 | 7.18 | 0.64 | 2.20 |
| BRS RAISSA RES 1/3 NCS | 7.18 | 1.46 | 4.00 |
| MSOY 7908 RR | 7.12 | 0.69 | 2.20 |
| PI358318A | 7.09 | 0.96 | 3.40 |
| TMGER15 31786 | 7.09 | 0.55 | 1.60 |
| TC12-0-52.294 | 7.05 | 0.51 | 1.40 |
| TC12-0-51.186 | 7.03 | 1.02 | 3.00 |
| TC12-0-50.332/G027 | 7.02 | 1.04 | 3.30 |
| TC130-00.011 | 7.01 | 0.37 | 1.10 |
| P1C142-188.840 | 6.95 | 0.93 | 2.70 |
| L263/264 CT | 6.89 | 1.15 | 3.80 |
| BR05736154 | 6.84 | 1.36 | 3.10 |
| G004501413260 - A | 6.77 | 0.70 | 1.90 |
| TC12-1-47.590 | 6.71 | 0.36 | 1.20 |
| TC12-2-60.290 | 6.68 | 1.22 | 4.00 |
| PI189861 | 6.64 | 1.27 | 3.60 |
| L525 (T28) | 6.60 | 1.32 | 3.60 |
| L671 (T3) | 6.59 | 1.02 | 2.80 |
| FT-71 | 6.59 | 1.11 | 3.40 |
| TC12-0-46.100 | 6.56 | 0.34 | 1.20 |
| Pickett | 6.53 | 0.88 | 2.50 |
| L165 (T92) | 6.50 | 0.35 | 1.10 |
| PI88788 | 6.49 | 1.22 | 3.90 |
| TC12-0-52.024 | 6.49 | 0.38 | 1.00 |
| P1C142-195.052 | 6.48 | 0.67 | 1.60 |
| TC12-0-48.687 | 6.47 | 0.44 | 1.10 |
| TC12-0-52.213 | 6.43 | 0.79 | 2.30 |
| PI398637 | 6.43 | 0.94 | 2.60 |
| P1C142-185.974 | 6.41 | 0.57 | 1.60 |
| TC12-0-52.294/G026 | 6.37 | 0.38 | 1.20 |
| TC12-0-51.331 | 6.37 | 0.92 | 3.30 |
| P1C142-170.329 | 6.27 | 0.43 | 1.20 |
| P1C142-170.215 | 6.26 | 0.49 | 1.30 |
